# Single‐Cell RNA Sequencing Reveals the Therapeutic Effects of Electroacupuncture at BL23 on Hyperuricemia‐Induced Nephritis

**DOI:** 10.1155/ijog/2494933

**Published:** 2025-11-21

**Authors:** Yiyang Hu, Xueyan Song, Haichang Li, Songyun Zhao

**Affiliations:** ^1^ The First School of Clinical Medicine, Zhejiang Chinese Medical University, Hangzhou, Zhejiang Province, China, zcmu.edu.cn; ^2^ Key Laboratory of Chinese Medicine Rheumatology of Zhejiang Province, College of Basic Medical Science, Zhejiang Chinese Medical University, Hangzhou, Zhejiang Province, China, zcmu.edu.cn

**Keywords:** electroacupuncture, hyperuricemia, inflammation, renal injury, scRNA-seq

## Abstract

Hyperuricemia (HUA) is a metabolic disorder characterized by elevated serum uric acid (UA) level, which would trigger inflammatory processes contributing to kidney damage. Acupuncture stimulation of BL23, a therapeutic strategy in traditional Chinese medicine (TCM), has been reported to promote diuresis and suppress the immune system and seems to be efficacious in HUA. In this study, we aimed to investigate the effect of electroacupuncture (EA) at BL23 on HUA and HUA‐induced kidney inflammation and dysfunction in mice. Mice received EA once daily after being given intragastric potassium oxonate (500 mg/kg) and adenine (100 mg/kg). EA administration not only decreased the levels of serum UA, creatinine, blood urea nitrogen, urinary UA, and protein, along with increased urinary CREA excretion, but also decreased the inflammatory cytokines productions (IL‐1*β*, IL‐6, and TNF‐*α*) in serum. scRNA‐seq of treated kidneys revealed that EA at BL23 suppressed the NLRP3 inflammasome complex, the IL‐6/JAK/STAT3 signaling pathway, and the production of IL‐6 and IL‐1*β* in HUA mice. Western blot analyses verified that EA suppressed the HUA‐induced NLRP3 inflammasome activation by promoting autophagy. In conclusion, the study demonstrated that EA at BL23 exhibited anti‐HUA and nephroprotective effects by inhibiting both the NLRP3 inflammasome and the IL‐6/JAK/STAT3 signaling pathway, reducing renal inflammation and supporting its therapeutic potential for HUA‐associated kidney injury.

## 1. Introduction

Hyperuricemia (HUA) is a metabolic syndrome characterized by elevated serum uric acid (UA) level above 420 *μ*M in men and 300 *μ*M in women on nonidentical days [1]. HUA has become a globally prevalent condition, particularly in high‐ and middle‐income countries, with reported prevalence rates as high as 36% [2]. Emerging evidence suggests that HUA may elicit kidney damage including nephritis and fibrosis, impairing renal function [3]. In particular, UA acts as a danger signal and is capable of eliciting immune system activation and alters the characteristics of resident kidney cells toward a proinflammatory and profibrotic state at clinically relevant concentrations [4]. These findings have led to increased awareness of HUA as a potential and modifiable risk factor for initiating or exacerbating inflammatory processes contributing to kidney disease.

The NLR family pyrin domain containing 3 (NLRP3) inflammasome, a key sensor of inflammatory danger signals, has recently been shown to play a crucial role in the pathogenesis of renal inflammation triggered by soluble UA and UA crystals [5]. NLRP3 assembles with apoptosis‐associated speck‐like protein (ASC) to form cytosolic oligomers to trigger the autocatalytic activation of pro‐Caspase‐1 into Caspase 1. Subsequently, Caspase‐1 cleaves pro‐interleukin‐1*β* (pro‐IL‐1*β*) to produce mature IL‐1*β* [6]. In addition, in response to UA accumulation, interleukin‐6 (IL‐6) activates the Janus kinase 2/signal transducer and activator of transcription 3 (JAK2/STAT3) signaling pathway, which further triggers the expression of suppressor of cytokine signaling 3 (SOCS3), promoting inflammatory cascades [7]. Furthermore, proinflammatory cytokines, including TNF‐*α* and IL‐6, upregulate the expression of the NLRP3 inflammasome, amplifying inflammation responses. Meanwhile, autophagy has recently been reported to play a crucial role in regulating inflammation, which degrades the NLRP3 inflammasome, leading to reduced levels of proinflammatory cytokines [8]. More and more studies reported that autophagy plays a protective role in HUA. The inhibition of autophagy exacerbates renal injury in HUA mice, while the activation of autophagy such as rapamycin can alleviate HUA‐induced renal injury by reducing inflammatory responses [9, 10]. Notably, HUA suppresses autophagy while activating both the NLRP3 inflammasome and the JAK2/STAT3 pathway, which collectively contribute to renal inflammation and injury [11].

Despite significant progress made in the management of HUA over the past decades, current treatment options primarily rely on xanthine oxidase (XOD) inhibitors such as allopurinol (ALL) and febuxostat, as well as UA reabsorption inhibitors including benzbromarone and probenecid [12]. While these medications have positive results in lowering UA levels, they do not have a substantial impact on improving the kidney damage associated with HUA [13]. Additionally, the potential therapeutic side effects may hinder the long‐term use of the current countermeasures, including hepatotoxicity, cardiovascular disorder risk, and rash [14]. For example, ALL is associated with gastrointestinal disturbances, rash, and Stevens–Johnson syndrome, as well as a rare but potentially fatal adverse reaction—ALL hypersensitivity syndrome [15]. Probenecid may cause neurodegeneration [16]. Thusly, the market awaits novel efficient strategies to the treatment of HUA.

Acupuncture has a long history in traditional Chinese medicine (TCM) as a therapeutic strategy works by dredging meridians and activating qi and blood and has gained increasing acceptance and popularity worldwide in recent decades [17]. With increasing evidence of its clinical efficacy, acupuncture is now a widely practiced convenient and safe treatment modality within complementary and integrative medicine worldwide [18]. Clinical and experimental studies have shown the potential benefits of acupuncture on UA levels, kidney function, and pain control by regulating sympathetic nerves or bioactive substances [19–21], while its role in lowering HUA‐induced nephropathy remains uncovered.

Acupuncture points are distributed throughout the body. An appropriate acupoint selection plays an indispensable role in acupuncture treatment. Shenshu (BL23) is a back‐shu point called “Shenshu” or “Jinyu” in Chinese or Japanese traditional medicine, respectively, that is located two fingers laterally between the second and third lumbar spinous processes [22]. The acupuncture stimulation of BL23 has been reported to affect kidney function (e.g., diuretic effects) [23], modulate the immune system (e.g., by reducing inflammatory cytokines) [24], and influence cerebral function [23] and bone metabolism [25]. Eiko Murakami et al. have reported that acupuncture at BL23 performed on healthy adults can suppress immune responses by reducing serum TNF‐*α*, IL‐13, and GM‐CSF levels [22]. Okumura et al. have suggested that BL23 acupuncture reduces the CD4/CD8 lymphocyte ratio, increases natural killer cells, and activates T cells in mice [26].

In the present study, we investigated the effects of BL23 acupuncture on serum UA and renal function in a HUA mouse induced by potassium oxanate (PO) and adenine hydrochloride (Ad). We further applied scRNA‐seq to elucidate the therapeutic mechanism of BL23 acupuncture in HUA‐induced nephropathy and explored the potential association of this alteration with NLRP3 inflammasome and JAK2/STAT3 signaling pathways.

## 2. Materials and Methods

### 2.1. Chemicals and Reagents

PO and sodium carboxymethyl cellulose were purchased from Aladdin (Shanghai, China). Ad was purchased from Ameresco (Framingham, MA, United States). Benzbromarone (BBR) was purchased from Kunshan Longdeng Ruidi Pharmaceutical Co., Ltd. (Kunshan, China). Antibodies against ASC (#67824), P62 (#39749), LC3 (#4108), Cleaved‐Caspase1 (# 89332), and Pro‐Caspase‐1 (#83383) were from the Cell Signaling Technology (Massachusetts, United States). Antibodies against NLRP3 (#ab263899), GAPDH (#ab181602), and *β*‐actin (#ab8226) were purchased from the Abcam (Massachusetts, United States).

### 2.2. Animal Experiment

Male C57BL/6 J mice (8‐week‐old, 25 ± 2 g) were purchased from Shanghai SLAC Laboratory Animal Co. Ltd., and were maintained under specific pathogen‐free conditions of the Zhejiang Chinese Medical University laboratory animal research center. Mice were housed with free access to standard food and sterile water under a 12 h light/dark cycle environment. The system maintains a constant temperature (23^°^C ± 2^°^C) and humidity of 40%–60%. All animals were handled in accordance with the protocols approved by the Institutional Animal Care and Use Committee of Zhejiang Chinese Medical University (Approval No. IACUC‐20230123‐06).

The HUA nephropathy was induced in mice by intragastric administration of PO (500 mg/kg) and Ad (100 mg/kg), suspended in 0.8% sodium carboxymethylcellulose solution, once daily at 8 a.m. for 14 consecutive days. The control group was intragastrically administered an equal volume of 0.8% sodium carboxymethylcellulose solution. All the intragastric administration was maintained throughout the entire experimental period until the mice were sacrificed.

Successfully modeled mice were anesthetized with isoflurane gas and treated with electroacupuncture (EA) or BBR. A custom‐made bipolar conjoined needle was inserted vertically at Shenshu (BL23) points (location of BL23: 3 mm lateral to the midline below the second lumbar vertebra) with a depth of 3–4 mm. The electrical stimulation parameters were set to alternating dense‐disperse waves at 2/15 Hz, 2 mA, for 15 min per session. The BBR group was intragastrically administered 6.5 mg/kg/day BBR as a positive control. The intervention was administered once daily at 4 p.m. for another 14 consecutive days after the HUA nephropathy modeling.

### 2.3. Sample Collection

Blood was collected from the inner canthus of the eye before sacrifice, and serum was prepared by centrifugation at 3000 rpm for 15 min. Urine samples were collected from mice placed in individual metabolic cages without access to food or water for a period of 4–6 h. The freshly voided urine was immediately centrifuged at 3000 rpm for 10 min at 4°C to remove cellular debris and particulate matter. Kidneys were harvested and either fixed in 4% paraformaldehyde or stored in cold 1640 medium for subsequent experiment.

### 2.4. Single‐Cell Suspension Preparation

The kidney was cut into 1 mm^3^ pieces with a small scissor on ice, then incubated in 5 mL of digestion buffer containing 0.25 mg/mL liberase thermolysin high research grade enzyme and 50 *μ*g/mL DNase I at 37°C for 30 min, shaking gently twice during the period. Then the digestion was stopped with 5% FBS. The digested tissue was then passed through a 70‐*μ*m cell strainer into prechilled PBS twice on ice and the cells were palleted by centrifugation at 400 g at 4°C for 5 min. After centrifugation, the cell pellet was incubated with 3 mL of red blood cell lysis buffer on ice for 5 min and palleted by centrifugation on 400 g at 4°C for 5 min. Then, the cells were washed and resuspended in prechilled PBS twice and filtered through a 40‐*μ*m cell strainer to remove debris or cell aggregates. Finally, the cells were centrifuged at 400 g at 4°C for 5 min and resuspended in 500 *μ*L of prechilled PBS with 0.04% BSA, and the dead cells were removed by micro beads.

### 2.5. Biochemical Analyses

The concentrations of UA, creatinine (CREA), blood urea nitrogen (BUN), and urine protein were measured using an automatic biochemical analyzer (Hitachi 3100, Tokyo, Japan).

### 2.6. Histopathological analyses

Fixed kidneys were embedded in paraffin, sectioned at 5 *μ*m thickness, and stained with hematoxylin and eosin (H&E). Micrographs of the sections were captured using an Aperio ScanScope digital scanner.

### 2.7. Enzyme‐Linked Immunosorbent Assay

The levels of TNF‐*α*, IL‐6, and IL‐1*β* were measured using enzyme‐linked immunosorbent assay (ELISA) kits (Elabscience Biotechnology, Wuhan, China) according to the manufacturer’s instructions.

### 2.8. scRNA‐seq Assay

Single‐cell RNA‐seq libraries were prepared using 10× Genomics Chromium Single Cell 3 ^′^ Reagent Kits according to the manufacturer’s instructions. Briefly, cells were encapsulated into droplets at a targeted cell recovery of cells. After the reverse transcription step, emulsions were broken and barcoded cDNA was purified with Dynabeads, followed by PCR amplification. Amplified cDNA was then used for 3 ^′^ gene expression library construction. Fifty nanograms of amplified cDNA was fragmented and end‐repaired. DNA fragmentation was analyzed by Agilent Fragment Analyzer 5300, double‐size selected with SPRI select beads, and sequenced on an Illumina platform using 150 paired‐end reads at a coverage of 40,000 mean reads per cell.

### 2.9. scRNA‐seq data processing

Unique molecular identifier (UMI) counts were obtained by aligning FASTQ files to the mm10 reference genome through Cell Ranger software. Gene expression raw count matrices were transformed into a Seurat object by Seurat R package for downstream analyses. High‐quality cells were retained according to the following criteria: expressed genes between 200 and 5000, less than 10% mitochondrial transcript, and unique gene counts more than 500. The scRNA‐seq count matrix was normalized using NormalizeData function in Seurat with default parameters. The top 3000 variable genes were used to perform principal component analysis (PCA). R package Harmony was used to remove batch effects with default settings by iteratively corrected PCA embedding based on top 50 PCA components. Then, we used the first 30 components to perform unsupervised cell clustering and nonlinear dimensionality reduction by FindNeighbor and FindClusters with a resolution of 1. UMAP coordinates were generated using RunUMAP. Marker genes for each cluster were identified by FindAllMarkers using MAST test. Cell type annotation was performed following comparison with classical cell type markers.

### 2.10. Differential Expression Enrichment and Scoring Analyses

The differentially expressed genes (DEGs) between two samples for different cell types were identified with FindMarkers. |log2FC| > 1 and adj. *p* value < 0.05 were used as the cutoff criteria. GO and Hallmark gene set enrichment analyses were conducted on these DEGs using the enrichr function implemented in the clusterProfiler package, and terms with *p* values less than 0.05 were considered significant. GO and Hallmark gene set were collected from the Molecular Signatures Database. The activity of inflammation‐related pathways was scored using AUCell and GSVA.

### 2.11. Western Blot Analyses

Total protein was extracted from murine kidney using RIPA lysis buffer (Cat# R0020, Solarbio). Protein was quantified by BCA assay (Beyotime, # p0012). The extract was subjected to SDS‐PAGE and subsequently electroblotted onto PVDF membranes. The proteins were probed and incubated with the specific primary antibodies, including NLRP3 (1:1000), ASC (1:1000), Pro‐Caspase 1 (1:1000), Cleaved‐Caspase 1 (1:1000), P62 (1:1000), LC3 (1:1000), *β*‐actin (1:2000), and GAPDH (1:10000) at 4°C overnight.

Then, the membranes were incubated with Goat anti‐Rabbit IgG(H + L)Secondary antibody (Thermo Pierce #31210) or Goat anti‐Mouse IgG(H + L)Secondary antibody (Thermo Pierce #31160) at room temperature for 2 h. Protein signals were visualized with ECL system, and the gray value was quantified as a ratio to *β*‐actin or GAPDH using ImageJ software.

### 2.12. Statistical Analyses and Visualization

Data are shown as mean ± SEM unless otherwise indicated. Statistical differences were analyzed by using a two‐tailed unpaired Student *t* test, one‐way or two‐way ANOVA with GraphPad Prism. If ANOVA was significant, individual differences were determined with Tukey post‐test. Differences were considered significant at the level *p* < 0.05. Graph generation were performed in GraphPad Prism and R. Visualization of gene expression by scatter plots, dot plots, and volcano plots were implemented through function “FeaturePlot()”, “DotPlot()”, “VlnPlot()” in the Seurat package, and “ggplot()” in the ggplot2 package. The UMAP graphs were plotted by “DimPlot()” function.

## 3. Results

### 3.1. EA Ameliorated HUA‐Induced Renal Injury

UA, a key indicator of HUA, is primarily excreted by the kidneys. Impaired excretion of UA can lead to kidney damage. As shown in Figure 1a, treatment with PO and Ad significantly elevated the serum UA level compared with the control group, indicating that the HUA model was successfully established. Similar to the positive control BBR, EA treatment significantly decreased the serum UA level compared with the HUA group. CREA and BUN are important indicators of renal function. Their levels were significantly elevated in the HUA group compared with the control group, indicating HUA‐induced kidney injury. Similar to the positive control BBR, EA treatment significantly suppressed the increasement of serum CREA and BUN induced by HUA. In addition to serum biochemical parameters, urinary biochemical assays are crucial for evaluating renal function in HUA mice. As shown in Figure 1b, the levels of urine UA, protein, and CREA were significantly elevated in the HUA group compared with the control group. Both EA and BBR treatments significantly reduced these elevated levels compared with the HUA model. Given that the inflammatory changes and structural alterations are pathological features of HUA, we further conducted renal histological examination. The H&E staining showed a normal morphology with no evidence of inflammation in the control group. While the mice after HUA modeling displayed a disorganized renal microstructure, characterized by thickened glomerular basement membrane, tubular dilatation, interstitial edema, and infiltration of inflammatory cells (Figure 1c). As expected, EA treatment remarkably ameliorated these pathological alterations in renal tissues of HUA mice, and so did BBR treatment. Collectively, these findings suggest that EA can effectively alleviate HUA‐induced kidney injury in mice.

Figure 1EA exerts a protective effect against HUA‐induced renal injury. At the end of the experiment, (a) serum UA, BUN, CREA and (b) urine UA, protein, CREA were examined. (c) Representative photographs of H&E‐stained kidney sections at 200× magnification. *n* = 6 mice per group.  ^∗∗^ *p* < 0.01,  ^∗∗∗∗^ *p* < 0.0001.

**Figure:**
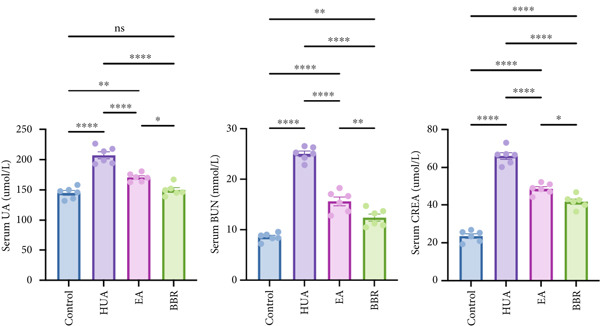
(a)

**Figure:**
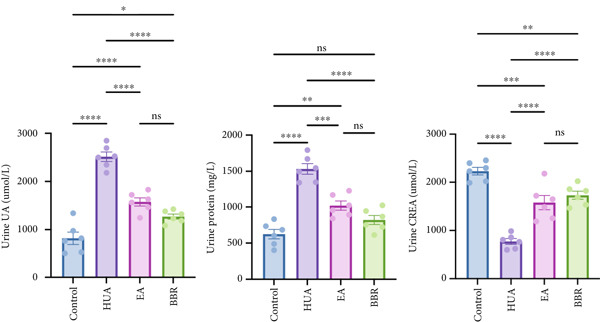
(b)

**Figure:**
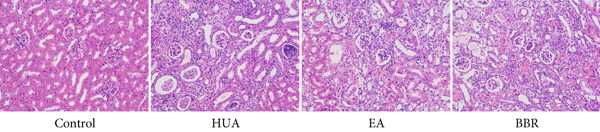
(c)

### 3.2. EA Attenuates Serum Inflammation Responses in HUA Mice

**Figure 2:**
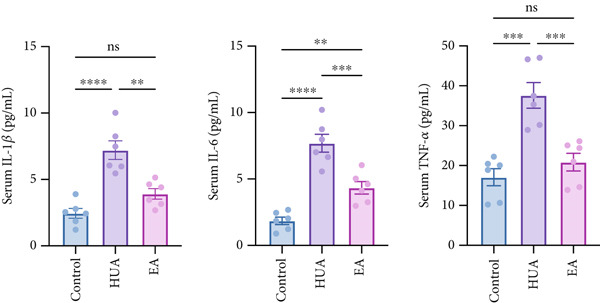
EA exerts an anti‐inflammation effect in HUA. At the end of the experiment, serum IL‐1*β*, IL‐6, and TNF‐*α* were examined. *n* = 6 mice per group.  ^∗∗^ *p* < 0.01,  ^∗∗∗∗^ *p* < 0.0001.

Since sustained elevation of UA may lead to inflammation, we next measured the serum levels of proinflammatory cytokines. As shown in Figure 2, the concentrations of IL‐1*β*, IL‐6, and TNF‐*α* were significantly increased after HUA modeling but decreased following EA treatment compared with the HUA group, suggesting that EA can attenuate HUA‐induced serum inflammatory responses.

### 3.3. Single‐Cell Sequencing and Cell Type Identification

Next, we performed scRNA‐seq of the kidneys from these mice. After quality control and removal of the batch effect between batches, 18659 single cells were identified into 10 clusters using unsupervised clustering (uniform manifold approximation and projection [UMAP]) (Figure 3a). Cluster‐specific genes were used to annotate cell types: macrophages (C1qa, C1qb, Ms4a7), endothelia cells (Emcn, Adgrl4, Sox17), fibroblasts (Col1a1, Dcn, Svep1), neutrophils (S100a8, S100a9, Clec4d), T cells (Cd3g, Trbc2, Tnfrsf9), epithelial cells (Cdh1, Epcam, Umod), mural cells (Acta2, Myh11, Rgs5), proximal tubule cells (Kcnj15, Slc27a2, Pck1), dendritic cells (Tspan13, Tmpo, Tbc1d4, Pfkp, Fgr), and B cells (Cd79a, Igkc, Ighm) (Figure 3b). To define the major cell‐type changes in HUA mice after EA treatment, the numbers and percentages of each cell cluster were compared between the two groups (Figure 3c,d). The proportions of macrophages, neutrophils, T cells, proximal tubule cells, and B cells decreased, while those of endothelial cells, fibroblasts, epithelial cells, mural cells, and dendritic cells increased in the EA group compared with the HUA group, suggesting that EA treatment may attenuate immune infiltration in the kidneys of HUA mice.

Figure 3Cell diversity in mouse kidney cells delineated by single‐cell transcriptomic analysis. (a) All samples with different treatments were integrated into a single dataset and clustered using UMAP. Colors and labels indicate different cell types based on marker gene expression. (b) Dot plot shows the expression of marker genes for the identified cell types. (c) Number and (d) percentages of identified cell types in each sample.

**Figure:**
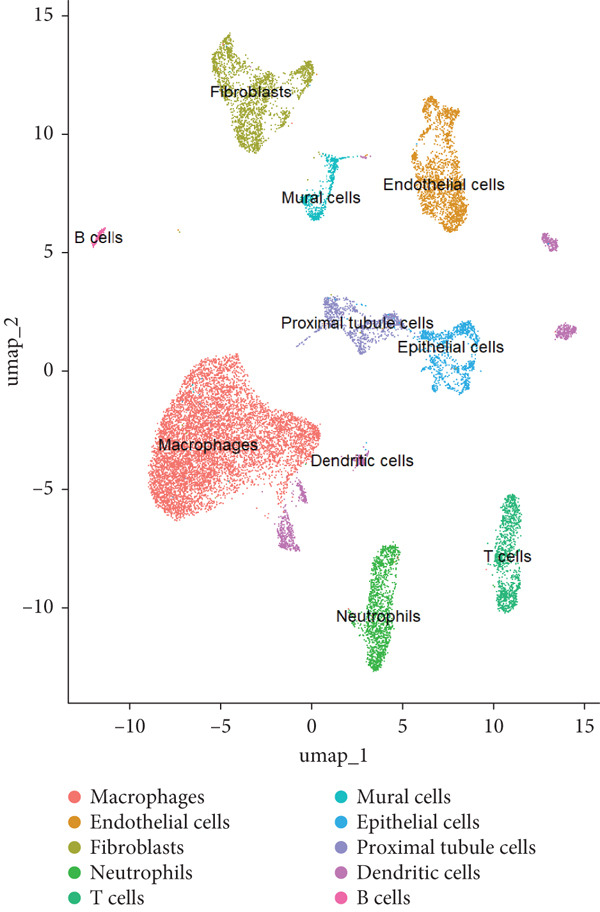
(a)

**Figure:**
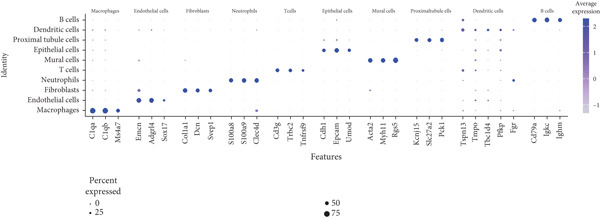
(b)

**Figure:**
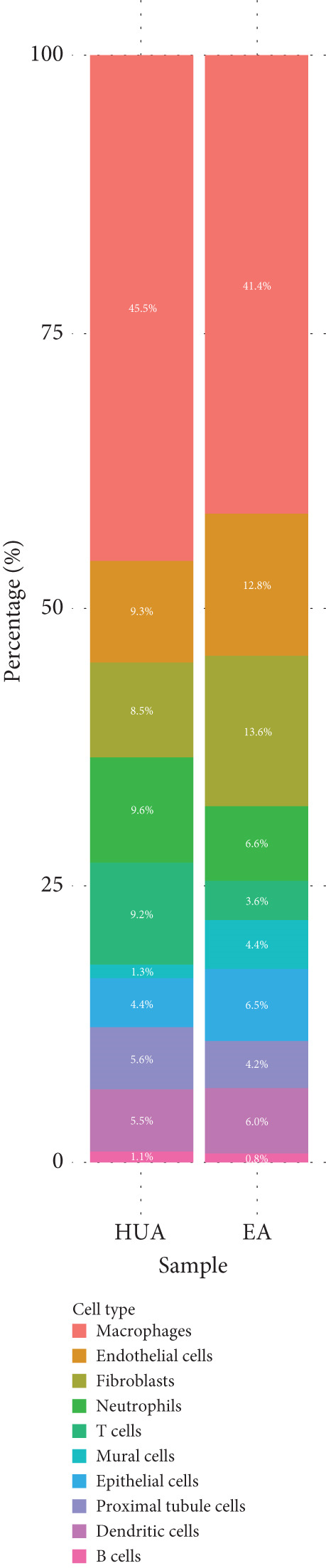
(c)

**Figure:**
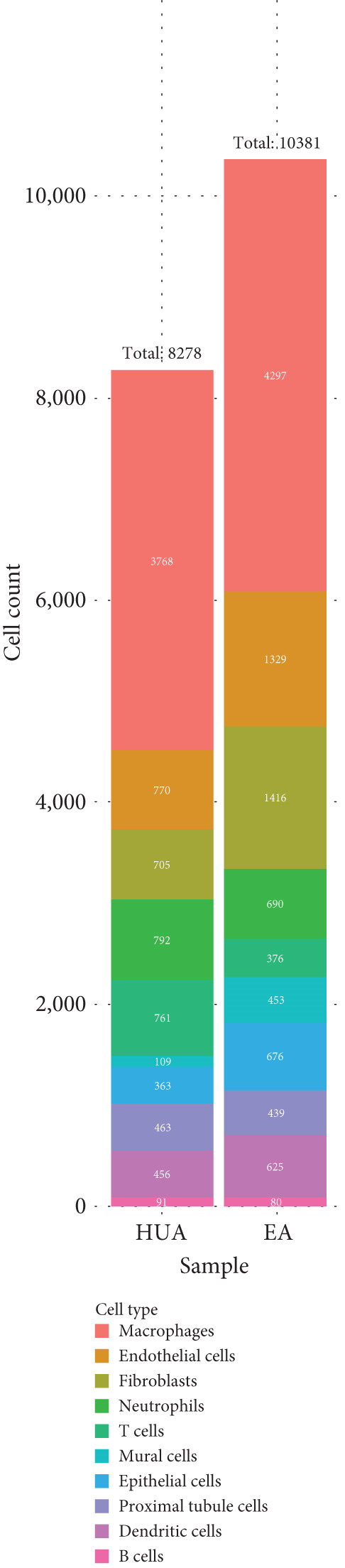
(d)

### 3.4. Differential Gene Expression and Pathway Enrichment Analysis

**Figure 5:**
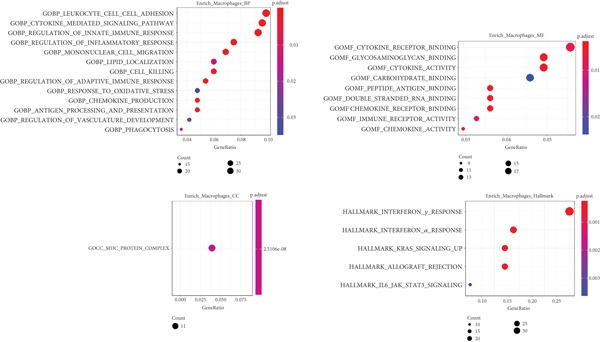
Functional enrichment analysis of DEGs in macrophages, based on GO biological process, cellular component, molecular function categories, and HALLMARK gene sets.

**Figure 6:**
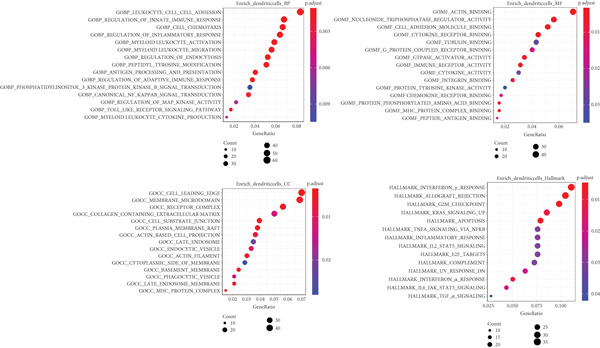
Functional enrichment analysis of DEGs in dendritic cells, based on GO biological process, cellular component, molecular function categories, and HALLMARK gene sets.

**Figure 7:**
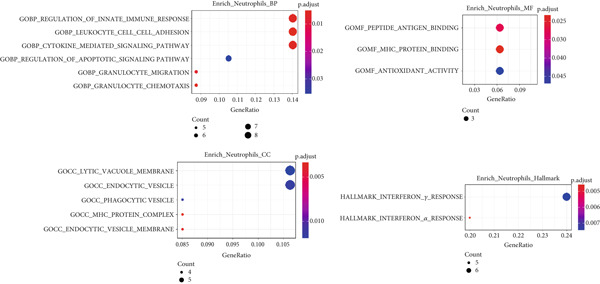
Functional enrichment analysis of DEGs in neutrophils, based on GO biological process, cellular component, molecular function categories, and HALLMARK gene sets.

**Figure 8:**
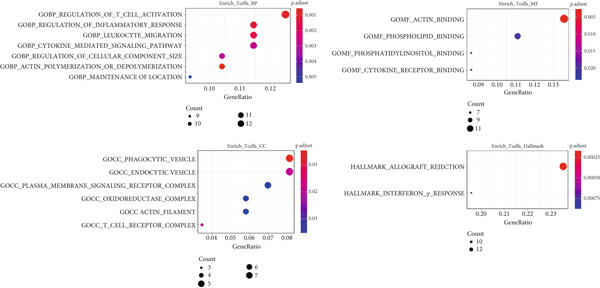
Functional enrichment analysis of DEGs in T cells, based on GO biological process, cellular component, molecular function categories, and HALLMARK gene sets.

Next, we identified the DEGs in all immune cell clusters and performed enrichment analysis in GO terms (BP, MF, CC) and Hallmark gene sets. In total, we identified 2987 DEGs in macrophages (557 upregulated and 2430 downregulated), 2023 DEGs in dendritic cells (366 upregulated and 1657 downregulated), 303 DEGs in T cells (13 upregulated and 290 downregulated), 197 DEGs in neutrophils (22 upregulated and 175 downregulated), and 5 DEGs in B cells (all downregulated) (Figure 4). The enrichment analysis revealed that DEGs in macrophages were mainly enriched in regulation of innate immune response, inflammatory response, and adaptive immune response (GO BP); cytokine receptor binding, immune receptor activity, cytokine activity, and chemokine receptor binding (GO MF); MHC protein complex (GO CC); as well as interferon gamma response and IL6–JAK–STAT3 signaling (Hallmark) (Figure 5). In dendritic cells, DEGs were primarily associated with the regulation of innate immune response, inflammatory response, and adaptive immune response (GO BP); cytokine receptor binding, immune receptor activity, cytokine activity, and chemokine receptor binding (GO MF); receptor complex, endocytic vesicle, phagocytic vesicle, and MHC protein complex (GO CC); and interferon gamma response, TNF*α* signaling via NF‐*κ*B, inflammatory response, and IL6–JAK–STAT3 signaling (Hallmark) (Figure 6). In neutrophils, DEGs were enriched in regulation of innate immune response and granulocyte migration (GO BP); MHC protein binding (GO MF); endocytic vesicle, phagocytic vesicle, and MHC protein complex (GO CC); and interferon gamma response (Hallmark) (Figure 7). In T cells, DEGs were mainly involved in regulation of T cell activation and inflammatory response (GO BP); cytokine receptor binding (GO MF); phagocytic vesicle, endocytic vesicle, and T cell receptor complex (GO CC); and interferon gamma response (Hallmark) (Figure 8). Enrichment analysis was not conducted for B cells because of the limited number of DEGs identified. Taken together, the results suggest that EA may exert regulatory effects on inflammatory and immune responses in HUA mice.

Figure 4Identification of DEGs between HUA and EA treatments. (a) Volcano diagram and (b) gradient volcano plot of DEGs across identified immune cells.

**Figure:**
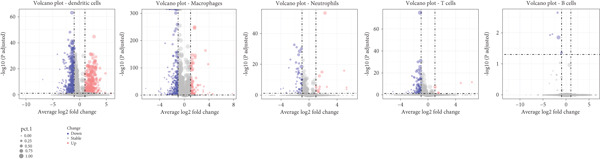
(a)

**Figure:**
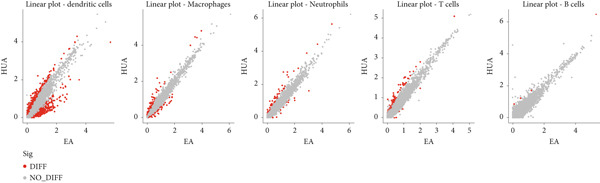
(b)

### 3.5. Inflammation‐Related Response Scores

**Figure 9:**
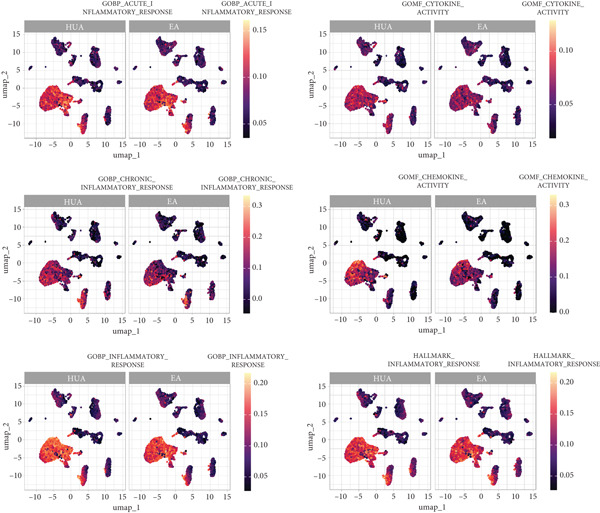
UMAP plots of single‐cell inflammation‐related response scores (AUCell) in the HUA and EA groups.

**Figure 10:**
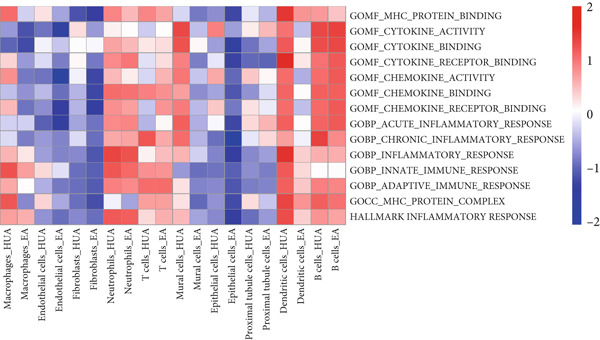
Heatmap of inflammation‐related response enrichment scores across different cell subpopulations between the HUA and EA groups, determined by GSVA. The color scale indicates the normalized GSVA score for each pathway.

Subsequently, inflammation‐related responses were assessed by AUCell, and the results showed that EA‐treated kidney exhibited attenuated inflammatory response including acute inflammatory response, chronic inflammatory response, cytokine activity, and chemokine activity compared with the HUA group (Figure 9). Consistent with previous findings, GSVA analysis validated that EA treatment could suppressed inflammation‐related responses across multiple cell types in HUA mice, including acute inflammatory response, chronic inflammatory response, cytokine activity, and chemokine activity (Figure 10).

### 3.6. Effects of EA on Modulating Inflammation‐Related Processes

**Figure 11:**
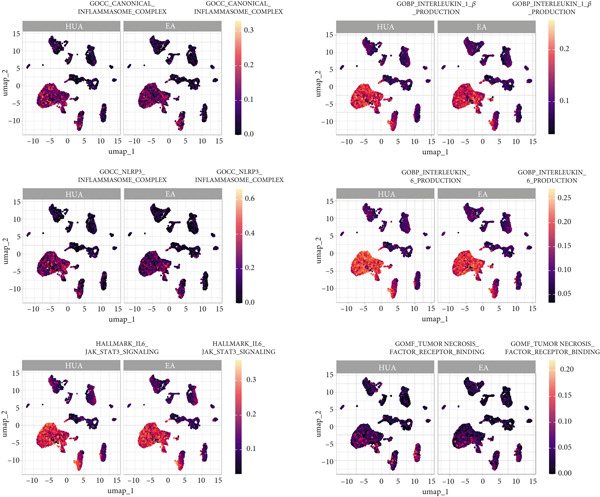
UMAP plots of single‐cell inflammation‐related process scores (AUCell) in the HUA and EA groups.

**Figure 12:**
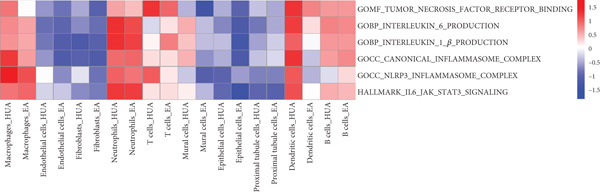
Heatmap of inflammation‐related process enrichment scores across different cell subpopulations between the HUA and EA groups, determined by GSVA. The color scale indicates the normalized GSVA score for each pathway.

**Figure 13:**
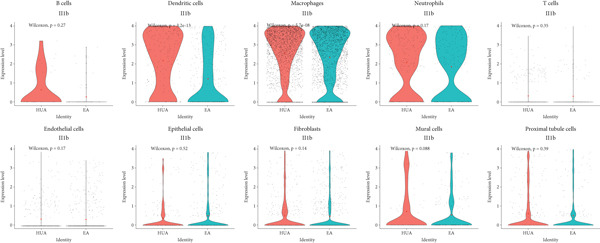
Violin plot of IL‐1*β* expression across different cell subpopulations between the HUA and EA groups. Each dot represents a single cell. The red diamond represents the average expression value for that group. Wilcoxon signed‐rank test was used to identify the statistic difference.

To elucidate the detailed mechanisms underlying the anti‐inflammatory effects of EA, we evaluated TNF*α*, IL‐1*β*, IL6, NLRP3, and the typical inflammatory mediators associated pathways using AUCell and GSVA scoring. As expected, six related gene sets (MF: TNF receptor binding; BP: IL‐6 and IL‐1*β* production; CC: canonical and NLRP3 inflammasome complexes; HALLMARK: IL6‐JAK‐STAT3 signaling) were downregulated in most cell types in the EA group compared with the HUA group (Figures 11 and 12). Meanwhile, the expression of IL‐1*β*, a key inflammation indicator, was observed to be downregulated in most cell types in the EA group compared with the HUA group, notably in dendritic cells, macrophages, and mural cells (Figure 13). Above all, scRNA‐seq analysis demonstrated that EA exerts anti‐inflammatory effects and reduces the IL‐1*β* expression in treating HUA‐induced nephropathy through downregulation of both the NLRP3 inflammasome and the JAK2/STAT3 signaling pathway.

### 3.7. EA Inhibits NLRP3 Inflammasome Activation in HUA Mice

**Figure 14:**
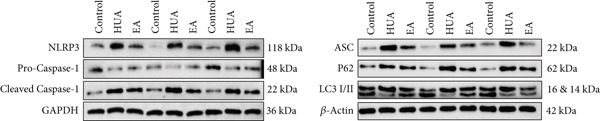
EA promotes autophagy and limits the activation of NLRP3 inflammasome in the kidneys of HUA mice. The western blot images of NLRP3, ASC, Pro‐Caspase 1, Cleaved‐Caspase 1, P62, LC3‐I, and LC3‐II.

To verify the hypothesis that EA alleviates renal inflammation induced by HUA through inhibition of the NLRP3 inflammasome, we analyzed the expression levels of NLRP3 pathway‐related proteins in kidney by western blot (Figure 14). The results showed that compared with the control group, the expression levels of NLRP3, ASC, and Cleaved‐Caspase 1 were increased while the level of Pro‐Caspase 1 was decreased in the kidneys of the HUA group. Notably, EA treatment effectively reversed these alterations, suppressing the levels of NLRP3, ASC, and Cleaved‐Caspase 1 and enhancing Pro‐Caspase 1 expression compared with the HUA group. These findings suggest that EA may alleviate inflammation responses in HUA mice by inhibiting the activation of the NLRP3 inflammasome, which is consistent with the single‐cell sequencing data. By eliminating misfolded proteins and inflammatory ligands, autophagy serves as a negative regulator of immune inflammation, capable of inhibiting NLRP3 inflammasome activation and the secretion of mature IL‐1*β*. To investigate the role of autophagy regulation in the anti‐inflammatory effects of EA, we measured the expression levels of key autophagy markers (LC3‐I, LC3‐II, and p62) in the mouse kidneys (Figure 14). Western blot analysis revealed a downregulation of LC3‐II/I but an upregulation of P62 in the kidneys of HUA mice compared with the control group, indicating the suppression of renal autophagy. Moreover, EA intervention increased the expression of LC3‐II/I and decreased the expression of P62, suggesting that its anti‐inflammatory effect is mediated through the promotion of autophagy, leading to suppression of NLRP3 inflammasome activation.

## 4. Discussion

HUA has emerged as a significant public health concern in the past decades across numerous countries [2]. Patients with HUA have elevated serum UA, which leads to renal inflammation, impairs kidney function, and in turn reduces UA excretion, further worsening both HUA and renal injury [3]. Globally, there has been some advancement in the study and creation of medications in urate‐lowering therapy (ULT). Clinical studies have been conducted on a number of new urate transporter 1 (URAT1) inhibitors. However, they can result in renal stones, similar to benzbromarone [27]. Although the identification of recombinant uricase offers us a fresh perspective, the recommended dosage is still low because of the possibility of permanent joint injury [28]. Despite the possibility of liver damage, febuxostat is still the first‐line ULT medication [28]. It will take time and effort to discover efficient medications to ULT in safety while minimizing renal damage.

Acupuncture has a long history in TCM as a safe complementary and integrative therapeutic strategy works and gained increasing acceptance and popularity worldwide in recent decades [17]. In this study, we found that EA at BL23 not only reduced serum UA levels but also ameliorated kidney dysfunction in HUA mice. We also identified a number of cellular subpopulations in kidney of HUA mice by using scRNA‐seq to elucidate the nephroprotective effects of EA at BL23.

HUA, characterized by elevated serum UA, occurs when UA is overproduced or under excreted due to disorders of the modulating system of UA [2]. Accumulating studies have shown that the sustained serum HUA may cause renal injury characterized by a dysregulated metabolism of CRE and BUN, the most important biochemical indexes to detect abnormal renal function [4]. The present study revealed that EA at BL23 ameliorated impaired renal function, as evidenced by decreased levels of serum UA, CREA, and BUN, along with reduced urinary UA and protein levels and increased urinary CREA excretion. Renal histopathological examination further validated that EA at BL23 ameliorates HUA‐induced kidney injury.

HUA is often accompanied by inflammatory storms, which burden kidney metabolism, as seen in increasing level of renal function biomarkers (BUN and CRE) and significantly renal injury [3]. Thus, the effective intervention of the inflammatory response is considered crucial in preventing the development of HUA. The inflammation responses that caused by sustained high serum UA level, induce an increase in proinflammatory factors including TNF‐*α*, IL‐1*β*, and IL‐6, and further aggravates renal injury, which is consistent with the present study [7]. EA at BL23 could decrease the abovementioned serum cytokines in HUA mice, suggesting that EA at BL23 can attenuate HUA‐induced serum inflammatory responses.

scRNA‐seq was further performed to examine the inflammatory response in the kidneys of HUA mice. We identified 10 cell clusters including macrophages, endothelia cells, fibroblasts, neutrophils, T cells, epithelial cells, mural cells, proximal tubule cells, dendritic cells, and B cells and found that the proportions of the main inflammation indicators (macrophages, neutrophils, T cells, B cells), and proximal tubule cells decreased, while those of endothelial cells, fibroblasts, epithelial cells, mural cells, and dendritic cells increased in the EA group compared with the HUA group, suggesting that EA at BL23 may attenuate immune infiltration in the kidneys of HUA mice.

There is a strong inflammatory response in the process of HUA, while dysregulation of immune cells, including dendritic cells, macrophages, neutrophils, and T cells, plays a crucial role in the inflammatory response [29]. We further identified the DEGs in all abovementioned cell clusters and performed enrichment analysis in GO terms (BP, MF, CC) and Hallmark gene sets. The DEGs were predominantly enriched in pathways related to inflammation and immunity, including regulation of innate and adaptive immune responses, inflammatory response, cytokine and immune receptor activity, IL‐6–JAK–STAT3 signaling, and TNF*α*–NF‐*κ*B signaling. AUCell and GSVA analysis was also performed on the abovementioned pathway, demonstrating that EA at BL23 could suppress inflammation‐related responses in HUA mice.

Considerable evidence suggests that HUA‐induced renal inflammation is closely related to the NLRP3 inflammasome activation, which upregulate the expressions of proinflammatory cytokines [30]. It has been reported that increased expressions of IL‐1*β* is closely associated with activation of the NLRP3 inflammatory pathway [11]. The previous studies also found that UA could induce activation of caspase 1 and release of IL‐1*β*, which in turn acts as an initiation signal for NLRP3 and propagates inflammatory responses [11, 31]. Moreover, HUA‐induced NLRP3 activation in macrophages is used as the contributors to the progression of diabetic nephropathy [32]. The elevated IL‐6 activates the JAK/STAT3 signaling pathway, which was also demonstrated in the HUA mouse [11]. Accumulating evidence suggests that the limitation of the NLRP3 inflammasome and JAK2/STAT3 signaling contributes to attenuate kidney inflammation [33, 34]. Similarly, our study showed that EA at BL23 suppressed the NLRP3 inflammasome complex, the IL‐6/JAK/STAT3 signaling pathway, and the production of IL‐6 and IL‐1*β* in the kidneys of HUA mice.

In order to further validate the transcriptional findings from single‐cell RNA sequencing, we examined the expression of the aforementioned inflammation‐related proteins in the kidneys of HUA mice. Western blot analyses revealed that EA suppressed the activation of the NLRP3 inflammasome pathway in HUA mice, as evidenced by reduced levels of NLRP3, ASC, and Cleaved‐Caspase 1, and promoted the induction of autophagy, a negative regulator of NLRP3 inflammasome, as evidenced by increased levels of LC3‐II/I and reduced levels of P62. Consistent with our results, both Wu et al.’s and Li et al.’s study reported that the inhibition of autophagy may lead to increased ROS accumulation, upregulated IL‐1*β* expression, and worsened kidney injury in HUA mice, whereas the activation of autophagy or inhibition of mitochondrial fission confers renal protection [9, 35].

In summary, this study performed scRNA‐seq on the kidneys of HUA mice and demonstrated that EA at BL23 alleviated HUA‐induced nephropathy by exerting anti‐inflammatory effects inhibiting the secretion of TNF‐*α*, IL‐1*β*, and IL‐6, through the downregulation of both the NLRP3 inflammasome and the IL‐6/JAK/STAT3 signaling pathway.

**Nomenclature**
Ad: adenine hydrochloride
ALL: allopurinol
ASC: apoptosis‐associated speck like protein
BP: biological process
BUN: blood urea nitrogen
CC: cell component
CREA: creatinine
DEGs: differentially expressed genes
EA: electroacupuncture
GO: Gene Ontology
H&E: hematoxylin and eosin
HUA: hyperuricemia
IL‐1*β*
: interleukin‐1*β*
IL‐6: interleukin‐6
JAK2: Janus kinase 2
MF: molecular function
NLRP3: NLR family pyrin domain containing 3
PCA: principal component analysis
PO: potassium oxanate
SOCS3: suppressor of cytokine signaling 3
STAT3: signal transducer and activator of transcription 3
TCM: traditional Chinese medicine
TNF‐*α*
: tumor necrosis factor‐*α*
UA: uric acid
ULT: urate‐lowering therapy
UMAP: uniform manifold approximation and projection
UMI: unique molecular identifier
URAT1: urate transporter 1
XOD: xanthine oxidase

## Ethics Statement

The animal study was reviewed and approved by the Laboratory Animal Management and Welfare Ethical Review Committee of Zhejiang Chinese Medical University.

## Conflicts of Interest

The authors declare no conflicts of interest.

## Author Contributions

YH and XS contributed equally to this work.

## Funding

This study is supported by National Key Research and Development Program of China, 10.13039/501100012166, 2022YFC3501204; Key Research and Development Program of Zhejiang Province, 10.13039/100022963, 2023C03040.

## Data Availability

The data that support the findings of this study are available from the corresponding author upon reasonable request.
